# Navigating the integration of large language models in healthcare: challenges, opportunities, and implications under the EU AI Act

**DOI:** 10.1186/s44158-024-00215-w

**Published:** 2024-12-02

**Authors:** Elena Bignami, Michele Russo, Roberto Lanza, Valentina Bellini

**Affiliations:** Anesthesiology, Critical Care and Pain Medicine Division, Department of Medicine and Surgery, University of Parma, Parma, Italy

## Introduction

In the rapidly evolving field of artificial intelligence (AI), large language models (LLMs) have emerged as powerful tools with transformative potential across healthcare sectors. These technologies promise enhanced diagnostic capabilities, patient education, and operational efficiencies. However, their integration into clinical practice is not without challenges, particularly in the context of stringent regulatory frameworks like the European Union’s AI Act [1, 2]. This editorial explores the juxtaposition of innovation and regulation, offering insights into how healthcare professionals can navigate these dynamics responsibly.

## Discussion

### Transformative potential of LLMs in healthcare

LLMs are increasingly recognized for their ability to process vast datasets and generate human-like text, with applications spanning medical diagnostics, administrative tasks, and patient engagement [3]. From simplifying radiology reports to drafting discharge summaries, these tools streamline workflows while improving patient comprehension [4, 4–7]. Furthermore, they hold promise in drug discovery and personalized medicine, fostering innovation at an unprecedented scale [8].

LLMs can also democratize access to healthcare knowledge. By generating plain-language explanations of complex medical concepts, these models empower patients and support clinicians in underserved areas. In resource-limited settings, LLMs can act as an accessible adjunct to healthcare professionals, mitigating the challenges of staffing shortages and expertise gaps [9].

However, these advancements must be balanced against inherent limitations. Their reliance on training datasets raises concerns about the quality, representativeness, and biases of the data, which can significantly impact outcomes. For instance, incorrect or oversimplified outputs may erode trust in healthcare systems or lead to adverse clinical decisions, underscoring the need for rigorous validation [10, 11].

### The EU AI Act: a regulatory milestone

The European Union’s AI Act, poised for adoption in 2024, establishes a comprehensive regulatory framework for AI systems, categorizing them by risk level—unacceptable, high, and limited. For healthcare, this risk-based approach translates into heightened scrutiny of AI applications in critical areas like diagnostics and treatment planning [2].

#### Key provisions and their implications

##### Risk categorization and transparency

The Act mandates transparency for limited-risk systems and stringent requirements for high-risk applications. For healthcare providers, this ensures that AI tools are used with informed oversight, fostering trust among clinicians and patients.

##### Prohibition of high-risk systems

AI systems deemed unacceptable, such as those involving biometric categorization or workplace emotion recognition, are explicitly banned. This safeguards fundamental rights and aligns with ethical principles integral to medical practice.

##### Governance and accountability

The establishment of regulatory sandboxes facilitates innovation while ensuring compliance. These controlled environments allow for the testing of AI tools in real-world scenarios, providing valuable insights without compromising ethical standards.

### Ethical and legal challenges

While the AI Act offers a structured approach, its implementation poses significant challenges. Key issues include the following:

i. Data privacy: LLMs process large datasets that may inadvertently include sensitive patient information, raising questions about data security and consent. Compliance with General Data Protection Regulation (GDPR) and related legislation are critical.
ii. Bias and equity: Models trained on skewed datasets risk perpetuating healthcare disparities. Proactive measures are required to identify and mitigate such biases, particularly when deploying LLMs in multicultural settings.
iii. Intellectual property: The ownership of outputs generated by LLMs remains a contentious issue, particularly in collaborative medical research where authorship and credit must be carefully managed.

### OpenAI’s privacy policies and healthcare considerations

The EU AI Act necessitates a revaluation of data handling practices by entities like OpenAI. While its privacy policies address general principles, greater specificity is required for high-risk sectors such as healthcare [12]. For instance as follows:

i. Handling sensitive data: Clear guidelines on managing healthcare data are essential to ensure compliance with GDPR and other local regulations.
ii. Transparency and user awareness: OpenAI must enhance disclosures regarding AI-generated content, particularly in clinical contexts where decisions may significantly impact patient outcomes.
iii. Mitigation of risk: OpenAI should consider developing healthcare-specific safeguards, including limitations on the use of LLMs in critical care decisions without clinician oversight.

### Fostering trust and collaboration

For LLMs to be effectively integrated into healthcare, fostering trust among all stakeholders is paramount. Patients, clinicians, and policymakers must be confident that AI tools are both safe and beneficial. This necessitates the following:

i. Patient-centric AI: Models must prioritize patient welfare, including clear communication of their limitations and a robust mechanism for addressing errors.
ii. Interdisciplinary collaboration: A collaborative approach involving engineers, ethicists, clinicians, and legal experts will ensure that LLMs are developed and deployed with comprehensive oversight.
iii. Global standards: The fragmented nature of AI regulation highlights the need for international harmonization. As the EU leads with the AI Act, other nations must align their frameworks to ensure consistency and interoperability.

### Balancing innovation and responsibility

The integration of LLMs in healthcare exemplifies the tension between technological progress and ethical responsibility. To achieve a sustainable balance as follows:

i. Education and training: Healthcare professionals must be equipped with the skills to evaluate AI tools critically. Curricula should incorporate AI literacy, focusing on its applications, limitations, and ethical implications [13].
ii. Continuous evaluation: AI tools should undergo ongoing assessments to ensure they meet evolving regulatory standards and clinical needs.
iii. Encouraging innovation: Regulatory sandboxes and similar initiatives allow innovation to flourish while maintaining ethical oversight, creating a fertile ground for AI-driven advancements.

### The role of healthcare leaders

Leadership in healthcare will play a pivotal role in determining the trajectory of AI integration. By advocating for responsible innovation, healthcare leaders can influence policy development, foster interdisciplinary collaboration, and guide the ethical deployment of LLMs. Their voices are crucial in shaping a future where AI enhances, rather than replaces, the human touch in medicine [14] (Table 1).

**Table 1:** Summary table: integration of large language models in healthcare

Category	Key points
**Transformative potential**	Streamlines clinical workflows with tasks like medical document summarization and discharge summaries
	Enhances medical education and personalized care through rapid synthesis of literature and tailored health advice
	Supports multimodal LLMs (M-LLMs) for integrating text, images, and sensor data to improve diagnostic accuracy
**Challenges**	Concerns over data privacy, particularly handling sensitive patient information
	Risks of biased outputs from nonrepresentative datasets perpetuating healthcare inequities
	Issues with model reliability and interpretability in critical clinical decisions
**Ethical concerns**	Ensuring fairness and transparency in AI-generated content
	Addressing biases to prevent exacerbating existing disparities in healthcare delivery
	Safeguarding patient trust through robust ethical and regulatory oversight
**Role of EU AI Act**	Establishes a risk-based framework categorizing AI systems by risk (limited, high, and unacceptable)
	Mandates transparency and prohibits high-risk systems like biometric categorization
	Encourages innovation via regulatory sandboxes, balancing progress with safety
**Future directions**	Calls for interdisciplinary collaboration among clinicians, technologists, and ethicists
	Promotes continuous evaluation and refinement of AI models to align with evolving healthcare needs
	Advocates for global standardization of regulations for consistent AI governance

## Conclusion

LLMs present a dual-edged sword: immense potential to enhance healthcare delivery paired with challenges that demand meticulous oversight. The EU AI Act provides a robust framework, but its success hinges on collaborative efforts among stakeholders. As healthcare professionals, embracing these tools responsibly will ensure they augment, rather than undermine, clinical excellence.

## Data Availability

No datasets were generated or analysed during the current study.

## Abbreviations

AI: Artificial intelligence
LLMs: Large language models
EU: European Union
M-LLMs: Multimodal large language models
GDPR: General Data Protection Regulation
